# Translation, adaptation, and validation of Person-Centered Primary Care Measures for patients in family doctor contract services within mainland China

**DOI:** 10.1186/s12875-025-02796-z

**Published:** 2025-03-31

**Authors:** Yang Wang, Dehua Yu, Hua Jin

**Affiliations:** 1 Department of General Practice, Research Center for General Practice, Yangpu Hospital, School of Medicine, Tongji University, Shanghai, China; 2 Shanghai General Practice and Community Health Development Research Center, Shanghai, China

**Keywords:** Primary care, Quality measurement, Quality improvement, Patient reported experience measure, China

## Abstract

**Background:**

In the context of China’s health reforms aimed at strengthening primary care through the Family Doctor Contract Service Program, effectively measuring its functional features is paramount. This study seeks to translate, adapt, and validate the Person-Centered Primary Care Measure (PCPCM) for primary care patients enrolled in family doctor contract services in mainland China.

**Methods:**

Following the guidelines by Sousa and Rojjanasrirat, we translated and adapted the PCPCM into Simplified Chinese and evaluated its psychometric properties. A total of 583 patients enrolled in family doctor contract services from 10 primary care facilities in Shanghai, China, participated in the study. We assessed the structural validity, internal consistency, stability reliability, and criterion validity of the PCPCM-Simplified Chinese version in accordance with the practical guidelines developed by the Core Outcome Measures in Effectiveness Trials (COMET) initiative and the Consensus-based Standards for the Selection of Health Measurement Instruments (COSMIN) initiative.

**Results:**

The study led to the development of the PCPCM-Simplified Chinese version tailored for patients receiving family doctor contract services (PCPCM-SC-FDCS), specifically designed to address the needs of populations most closely aligned with the concept of “primary care patients” in mainland China. Initial pilot testing prompted refinements to enhance clarity and applicability, particularly for Item 5 (Relationship). Analyses of the refined PCPCM-SC-FDCS, based on a three-point Likert scale, revealed that structural validity, internal consistency, and criterion validity all met the criteria for good measurement properties outlined in the relevant guidelines. However, for test-retest reliability, the intraclass correlation coefficient (ICC) between the first and second surveys was 0.58, which fell short of the recommended threshold of ≥ 0.70.

**Conclusions:**

The PCPCM-SC-FDCS demonstrates satisfactory reliability and strong feasibility as a tool for evaluating the functional features of primary care among Family Doctor Contract Service Program patients in mainland China. Although further testing and refinement are necessary, this instrument offers a feasible and straightforward approach to evaluating service quality, supporting family doctor teams in enhancing primary care delivery.

**Supplementary Information:**

The online version contains supplementary material available at 10.1186/s12875-025-02796-z.

## Background

Primary care is pivotal in achieving enhanced outcomes in both population health and health equity, while simultaneously ensuring cost-effectiveness, relative to a specialist-centric healthcare model [1, 2]. Its efficacy is inherently tied to its distinctive functional features within healthcare services, such as accessibility, continuity, comprehensiveness, and coordination [2, 3]. This underscores the importance of assessing these features to evaluate performance and quality [4]. To achieve this, various instruments have been developed and empirically validated in different countries. Notable among these are the Primary Care Assessment Tool (PCAT), the Quality and Outcomes Framework (QOF), and the Person-Centered Primary Care Measure (PCPCM) [5].

A major challenge in assessing the functional features of primary care in Mainland China lies in its distinctive historical and societal background, which is closely intertwined with an evolving healthcare system under reform. Established between the 1950s and 1970s, China’s primary care system was initially designed to provide equitable and accessible health services to rural and economically disadvantaged populations [6]. However, this focus shifted during the market-driven healthcare reforms of the 1970–1990 s, which severely curtailed resources for primary care and significantly weakened its clinical capacity and scope of services. Consequently, an increasing number of patients began bypassing primary care facilities in favor of visiting specialty outpatient clinics at large general hospitals [7].

In response to these challenges, the Chinese government launched the Family Doctor Contract Service Program in 2009 as a key policy initiative to strengthen primary care [8]. Central to this program is the establishment of family doctor teams, which forge long-term therapeutic relationships with local community residents who voluntarily contract with them. Each team—designed to serve up to 2,000 individuals—is based in primary care facilities and typically includes primary care physicians (such as general practitioners and community specialist doctors), supported by nurses, physician assistants, and public health doctors [9]. These teams provide a comprehensive range of primary care services to their contracted residents, including health consultations, routine physical examinations, diagnosis and treatment of common ailments, chronic disease management, and traditional Chinese medicine services [8]. They are also responsible for delivering essential public health services in specific communities or villages, such as vaccinations, surveillance of certain infectious and non-communicable diseases, and preventive care for vulnerable populations including children, pregnant women, and the elderly [10]. In some regions, clinical capacity has been enhanced through increased medical resource allocation, such as expanded prescription authority, and regular outpatient services provided by specialist doctors from major hospitals at primary care facilities [8].

Although the program currently covers only about 30% of residents [11] and, due to specific policy designs, primarily includes individuals aged 65 and older as well as patients with hypertension or diabetes [8], the Chinese government has released official documents aiming to extend coverage to at least 75% of the population by 2035^11^. Consequently, the family doctor contract service program holds the potential to become a cornerstone for achieving universal healthcare coverage and delivering higher-quality primary care services across Mainland China.

We believe it is necessary to translate and adapt the PCPCM for use in evaluating the quality of the family doctor contract service program in China because it offers a distinct advantage over other instruments: it is firmly rooted in the doctor-patient therapeutic relationship [12] and its eleven items directly solicit patients’ experiences of essential beneficial aspects of primary care, rather than indirectly measuring these aspects through specific healthcare services [13]. This design enables a more flexible and accurate evaluation of the target construct across various social contexts and healthcare systems than methods that rely on fixed scenarios and limited populations within a specific healthcare setting. Additionally, the PCPCM comprises only 11 specific questions, far fewer than other scales with similar purposes that have been translated into Chinese and applied in mainland China’s primary care context, such as the full and simplified versions of the PCAT [14, 15], and General Practice Assessment Questionnaire (GPAQ) [16]. Given the heavy workload of family doctors, the brevity of the PCPCM promotes higher response rates, better patient cooperation, greater data reliability, and enhanced survey efficiency and feasibility.

Currently, while the PCPCM has been translated and validated in various languages across 35 Organisation for Economic Co-operation and Development (OECD) countries and regions [17], including Hong Kong [18, 19], the substantial linguistic differences between traditional and simplified Chinese, coupled with the significant disparities in the health systems of Hong Kong and mainland China^20^.^21^, highlight the need for a version specifically tailored to patients receiving family doctor contract services in mainland China. Thus, this study aims to translate, adapt, and validate the PCPCM into Simplified Chinese, ensuring it accurately captures patient experiences within this unique population.

## Methods

The original PCPCM consists of a question followed by 11 items. Respondents are prompted to answer using a four-point Likert scale, with options ranging from “Definitely” to “Not at all” [13]. In this study, we adhered to the cross-cultural translation, adaptation, and validation guidelines proposed by Sousa and Rojjanasrirat [22], as well as the practical guidelines developed by the Core Outcome Measures in Effectiveness Trials (COMET) initiative and the Consensus-based Standards for the Selection of Health Measurement Instruments (COSMIN) initiative regarding measurement properties [23]. A three-step process was employed to develop a Simplified Chinese version of the PCPCM for patients receiving family doctor contract services (PCPCM-SC-FDCS).

### Development of pre-final version of PCPCM-SC-FDCS

The initial phase in developing the PCPCM-SC-FDCS was grounded in a meticulous and culturally sensitive translation process. This phase involved two bilingual translators, both possessing fluency in English and Simplified Chinese. The first translator brought practical experience in general practice and public health, while the second translator was deeply familiar with the local primary care contexts and nuances of the family doctor contract service, although with a more limited background in health sciences. To ensure translation fidelity, these translators independently converted the PCPCM from English to Simplified Chinese. Special attention was given to aligning the translation with the specific needs and experiences of our target population—patients under family doctor contracts. Their independent translations were then scrutinized by a third bilingual translator, an active general practitioner in a Chinese primary care setting. This translator’s role was pivotal in identifying and resolving inconsistencies between the two translations, focusing on terminology, sentence structure, conveyed meaning, and the suitability of the adaptations made. Through collaborative efforts, these translations were harmonized, culminating in a preliminary version of the PCPCM-SC-FDCS.

Further refining the translation, two additional bilingual translators, both native English speakers studying in China, independently back-translated the preliminary version into English. One translator specialized in general practice, while the other, lacking a specialized background in health sciences, brought a different perspective. A comprehensive review session with all five translators was then organized. The insights gained from this session were instrumental in refining and finalizing the translation, culminating in the development of the pre-final version of the PCPCM-SC-FDCS.

### Pilot testing of PCPCM-SC-FDCS

The pilot testing of the pre-final version of the PCPCM-SC-FDCS was conducted in two phases. In the first phase, five family doctors from five provinces (Beijing, Chongqing, Henan, Liaoning, and Shanghai) recruited four patients each, all of whom were registered with their respective family doctors. These patients evaluated the clarity of the instructions, items, and response format of the translated scale using a simple dichotomous scale (clear or unclear). Advancement to the second phase of testing was contingent upon over 80% of participants reporting clarity in the instructions, response format, and individual items.

Additionally, we assembled a panel of 10 experts from the general practice departments of general hospitals and the public health departments of universities in mainland China, all of whom possess a solid understanding of the functional features of primary care. Following their feedback, we made meticulous adjustments to the wording, proceeding only after more than 80% of the panel agreed that the instructions, response format, and each item were sufficiently clear to confirm conceptual equivalence.

During the second phase, we engaged these ten experts to evaluate the content relevance of each item using a four-point scale, where 1 = not relevant, 2 = unable to assess relevance, 3 = relevant but requiring minor alteration, and 4 = very relevant and succinct. Items receiving a rating of 1 (not relevant) or 2 (unable to assess relevance) were further refined. To ensure robust content validity, we set a minimum threshold of Scale-Content Validity Index/Average (S-CVI/Ave) ≥ 0.90^22^.

### Psychometric testing of PCPCM-SC-FDCS

In August 2023, we recruited a total of 583 primary care patients from 10 primary care facilities in Shanghai, China, all of whom were enrolled in the Family Doctor Contract Service Program. These facilities were strategically selected to reflect diverse settings, including urban (4 facilities), suburban (3 facilities), and rural (3 facilities) areas. At each facility, with the assistance of a family doctor affiliated with the center, we employed convenience sampling to invite no more than 100 patients to participate. The selected patients were primarily those listed in the registration records and familiar with their family doctor. Participants completed the survey by accessing a link to an electronic questionnaire, designed using the “Survey Star (Wenjuanxing)” platform, on their mobile phones. For older adults unfamiliar with smartphone use, family doctors assisted in completing and submitting the questionnaire.

In accordance with established empirical standards, this sample size was considered adequate for the psychometric analysis of the eleven dimensions of the pre-final versions of the PCPCM-SC-FDCS [22]. The inclusion criteria included: (1) being aged 18 years or older; (2) cognitive alertness and ability to read or understand the relevant informed consent documents; (3) having utilized at least one primary care service from a family doctor in the past 12 months, consistent with the implementation recommendations for the target population set forth by the American Academy of Family Physicians [24].

In addition to the PCPCM-SC-FDCS, the survey collected data on participants’ gender, age, educational level, mean annual income, EuroQol-5 Dimensions (EQ-5D), duration of family doctor contracts, frequency of consultations with a family doctor in the past year, and responses to the full Chinese version of the PCAT. Two weeks after the initial survey, family doctors distributed a follow-up questionnaire containing only the PCPCM-SC-FDCS to the same participants and invited them to complete it.

Using the collected data, we employed descriptive statistics to summarize the sociodemographic characteristics of the surveyed patients. Subsequently, we conducted psychometric analyses of the PCPCM-SC-FDCS in accordance with guidelines jointly published by the COMET and COSMIN initiatives. These analyses evaluated the scale’s structural validity, internal consistency, stability reliability, and criterion validity. The specific analytical methods and judgment criteria are detailed in Table 1. All statistical analyses were performed using R version 4.3.2 (R Foundation for Statistical Computing, Vienna, Austria).

**Table 1:** Methodology for psychometric analysis of PCPCM-SC-FDCS

Measurement property	Psychometric analysis	Criteria for judgment
Structural validity	We conducted Rasch analysis to assess the structural validity of the PCPCM-SC-FDCS scale, examining unidimensionality, local independence, monotonicity, and overall model fit.	1. Unidimensionality: Standardized item-person fit residuals fall within the range of -2.5 to 2.5. 2. Local Independence: Q3 values are less than 0.37. 3. Monotonicity: The scalability coefficients of the items should be greater than 0.30 to ensure monotonicity in the measurement. 4. Model Fit: Infit and outfit mean squares fall within the range of 0.5 to 1.5.
Internal consistency	We assessed the internal consistency of the PCPCM-SC-FDCS scale by calculating Cronbach’s alpha and examining evidence for unidimensionality using PCA with the proportion of variance explained and the scree plot.	1. Cronbach’s Alpha: Values between 0.70 and 0.95. 2. Unidimensionality via PCA: The first principal component explains a substantial portion of variance (e.g., > 20%) and is supported by a clear “elbow” in the scree plot.
Reliability	We administered the questionnaire twice, 14 days apart, to the same cohort of patients enrolled in the family doctor contract service to evaluate test-retest reliability using ICC.	ICC was ≥ 0.70.
Criterion Validity	We evaluated the convergent validity of the PCPCM-SC-FDCS by comparing its scores to the widely adopted and validated Chinese version of the PCAT, currently the most classic and widely used instrument for assessing primary care functional features in mainland China.	The correlation with the PCAT was ≥ 0.70.

Note:

PCPCM-SC-FDCS: Person-Centered Primary Care Measure-Simplified Chinese Version for Family Doctor Contracted Patients

PCA: Principal Component Analysis

ICC: Intraclass Correlation Coefficient

PCAT: Primary Care Assessment Tool

### Ethics approval and consent to participate

#### Ethical approval

for this study was granted by the ethics review board at Peking University (IRB00001052-23077). Informed consent was obtained verbally from all participants, in line with the ethical permissions. In accordance with the permissions granted by the ethics committee, informed consent was obtained verbally from all participants. The participants were fully informed about the purpose of the study, their right to withdraw at any time without any consequences, and the measures taken to ensure their privacy and the confidentiality of their responses. Verbal consent was deemed appropriate by the ethics committee due to the nature of the study and was documented accordingly.

## Results

Through the collaborative efforts of five translators and feedback from 20 primary care patients and 10 experts, the PCPCM was translated and adapted into the PCPCM-SC-FDCS (see Table S1). The most significant revision involved Item 5 (Relationship), originally phrased as “My family doctor or the team could coordinate the care I get from multiple places.” This was updated to “My family doctor or the team is aware of the medical services I receive from various institutions.” This revision addressed concerns from 70% of experts and 50% of patients, who noted the limited applicability of care coordination in mainland China’s primary care due to the lack of robust gate-keeping systems and formal referral mechanisms. After these revisions, over 80% of experts and patients found the content clear. The Scale Content Validity Index/Average (S-CVI/Ave) was calculated at 0.90, enabling the study to proceed to psychometric analysis (see Table S2).

Among the 583 patients surveyed, 466 (79.9%) had hypertension or diabetes, and 411 (70.5%) were aged 65 or older. Additionally, 69.5% of respondents had been enrolled in the Family Doctor Contract Service Program for more than three years, and 78% had consulted their family doctor three or more times in the past year. Other sociodemographic characteristics are presented in Table 2. Respondents reported high levels of primary care functionality, as measured by the PCPCM-SC-FDCS and PCAT during the first survey and by the PCPCM-SC-FDCS during the second survey. The average scores were 3.67, 3.59, and 3.61, respectively, on a 4-point Likert scale, with 4 indicating the highest functionality (see Table 3).

**Table 2:** The sociodemographic characteristics of surveyed primary care patients enrolled in family Doctor contract services in Mainland China

Characteristic	All patients
Gender(%)	
Male	218(37.39)
Female	365(62.61)
Age (Mean ± SD, Years)	67.57(0.82)
Highest Education Completed (%)	
Did Not Complete Primary School	16(2.74)
Completed Primary School	93(15.95)
Completed Junior Middle School	192(32.93)
Completed High School	165(28.30)
Completed College or Higher	117(20.07)
Annual Household Income (Mean ± SD, Yuan)	64127.05(45523.34)
EQ-5D Utility Index (Mean ± SD, Scores)	0.93(0.16)
Duration of family doctor contracts (%)	
Less than 1 year	45(7.72)
1–2 years	34(5.83)
2–3 years	99(16.68)
More than 3 years	405(69.47)
Frequency of consultations with a family doctor in the past year (%)	
1–2 Times	128 (21.96)
3–5 Times	131 (22.47)
6–10 Times	162 (27.79)
More than 10 Times	162 (27.79)

Note:

EQ-5D: EuroQol-5 Dimensions

**Table 3:** Patient experience for primary care patients enrolled in family Doctor contract services in Mainland China

Scale	Mean (SD)
PCPCM-SC-FDCS: Initial survey	
Item 1 (Accessibility)	3.83(0.41)
Item 2 (Comprehensiveness)	3.79(0.43)
Item 3 (Integration)	3.68(0.55)
Item 4 (Coordination)	3.61(0.63)
Item 5 (Relationship)	3.68(0.54))
Item 6 (Continuity)	3.45(0.71)
Item 7 (Advocacy)	3.67(0.56)
Item 8 (Family context)	3.64(0.59)
Item 9 (Community context)	3.58(0.61)
Item 10 (Health Promotion)	3.73(0.52)
Item 11 (Goal-oriented care)	3.70(0.50)
Total	3.67(0.45)
PCPCM-SC-FDCS: Follow-up survey	
Item 1 (Accessibility)	3.84(0.40)
Item 2 (Comprehensiveness)	3.73(0.52)
Item 3 (Integration)	3.61(0.62)
Item 4 (Coordination)	3.57(0.60)
Item 5 (Relationship)	3.57(0.61)
Item 6 (Continuity)	3.32(0.77)
Item 7 (Advocacy)	3.59(0.64)
Item 8 (Family context)	3.58(0.65)
Item 9 (Community context)	3.58(0.60)
Item 10 (Health Promotion)	3.67(0.54)
Item 11 (Goal-oriented care)	3.66(0.53)
Total	3.61(0.47)
PCAT	
First contact Utilization	3.74(0.41)
First contact access	3.31(0.43)
Ongoing care	3.53(0.36)
Coordination	3.68(0.29)
Coordination (information systems)	3.71(0.34)
Comprehensiveness	3.47(0.39)
Family-centeredness	3.75(0.37)
Community orientation	3.69(0.37)
Culturally competent	3.44(0.48)
Total	3.59(0.52)

Note:

PCPCM-SC-FDCS: Person-Centered Primary Care Measure-Simplified Chinese Version for Family Doctor Contracted Patients

PCAT: Primary Care Assessment Tool

Since no respondents selected “Not at all” for Items 1 and 2 (assessing accessibility and comprehensiveness), the response categories “Not at all” and “Somewhat” were merged. This adjustment transformed the 4-point Likert scale into a 3-point scale for subsequent psychometric analysis. The structural validity assessment indicated an overall Outfit Mean Square Error of 1.12 and Infit Mean Square Error of 1.02, both within the acceptable range of 0.5 to 1.5. Standardized item-person fit residuals ranged from − 1.92 to 1.52, within the permissible range of -2.5 to 2.5. The maximum adjusted Q3 value was 0.36, slightly below the upper limit of 0.37. Scalability coefficients of the items ranged from 0.64 to 0.72, exceeding the acceptable lower limit of 0.30 (See Table 4).

**Table 4:** Results of psychometric analyses of PCPCM-SC-FDCS

Psychometric analysis	Results	
Structural validity		
**Model Fit**	**Outfit MNSQ**	**Infit MNSQ**
Item1_Cat1^*^	1.24	1.32
Item1_Cat2	0.82	0.96
Item2_Cat1	1.31	1.39
Item2_Cat2	1.25	0.99
Item3_Cat1	1.59	0.92
Item3_Cat2	0.98	1.01
Item4_Cat1	1.34	1.07
Item4_Cat2	1.15	1.09
Item5_Cat1	1.05	1.02
Item5_Cat2	0.91	0.90
Item6_Cat1	1.27	1.26
Item6_Cat2	1.05	1.16
Item7_Cat1	1.47	0.90
Item7_Cat2	0.69	0.79
Item8_Cat1	1.34	0.93
Item8_Cat2	0.72	0.81
Item9_Cat1	1.35	0.95
Item9_Cat2	0.83	0.93
Item10_Cat1	0.98	1.04
Item10_Cat2	0.87	0.87
Item11_Cat1	1.43	0.95
Item11_Cat2	1.15	0.99
Overall Fit (Average)	1.12	1.02
Standardized item-person fit residuals (range):	-1.92 to 1.52.	
Maximum adjusted Q3 value	0.36	
Scalability coefficients (range)	0.64 to 0.72	
Internal consistency		
**Principal Component Analysis**	**Eigenvalue**	**Proportion of Variance**
Item 1	6.95	63%
Item 2	0.87	8%
Item 3	0.65	6%
Item 4	0.46	4%
Item 5	0.42	4%
Item 6	0.38	3%
Item 7	0.31	3%
Item 8	0.3	3%
Item 9	0.23	2%
Item 10	0.22	2%
Item 11	0.2	2%
Cronbach’s Alpha	0.94	
Stability reliability		
ICC	0.58	
Criterion validity		
Spearman correlation coefficient with the PCAT	0.74	

Note:

Outfit MNSQ: Outfit Mean Square Error

Infit MNSQ: Infit Mean Square Error

*: Cat (Category): indicates the response categories for each PCPCM-SC-FDCS item (e.g., 3-point Likert scale response options, where 0 = “Somewhat or Not at all,” 1 = “Mostly,” and 2 = “Definitely”)

PCPCM-SC-FDCS: Person-Centered Primary Care Measure-Simplified Chinese Version for Family Doctor Contracted Patients

ICC: Intraclass Correlation Coefficient

PCAT: Primary Care Assessment Tool

**Fig. 1:**
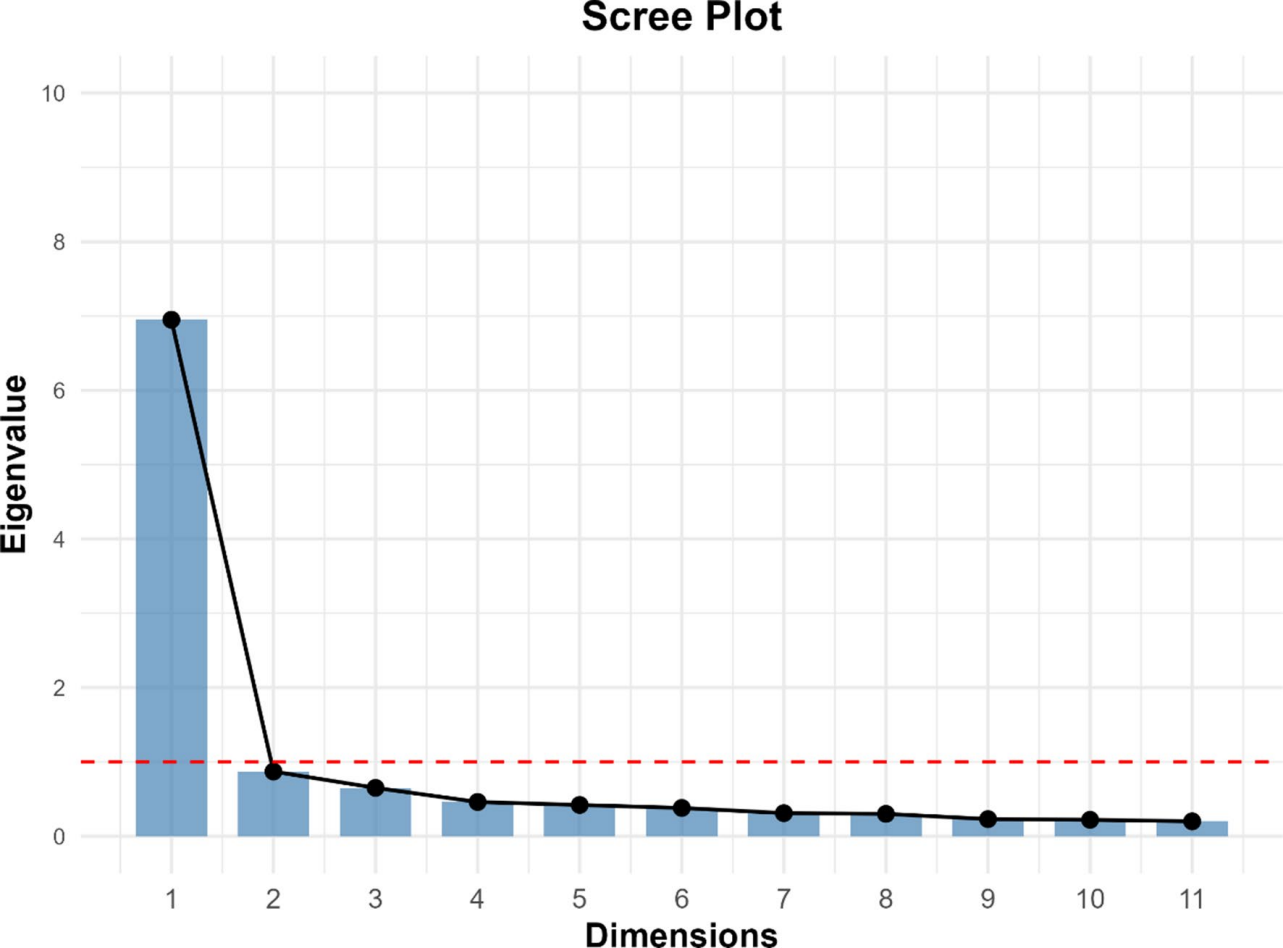
Scree plot: principal component analysis of the PCPCM-SC-FDCS. Note: The X-axis values from 1 to 11 correspond sequentially to the 11 items of the PCPCM-SC-FDCS

Following the structural validity assessment, the psychometric analysis proceeded to evaluate three additional properties, as detailed in Table 4. For internal consistency, principal component analysis revealed that the first principal component accounted for 63% of the total variance, indicating strong unidimensionality, further supported by the Scree Plot in Fig. 1. Cronbach’s alpha was 0.94, approaching but not exceeding the acceptable upper limit of 0.95. For criterion validity, the correlation coefficient between the PCPCM-SC-FDCS and the PCAT was 0.74, surpassing the recommended threshold of 0.7. However, for stability reliability, the intraclass correlation coefficient between the first and second surveys was 0.58, falling short of the recommended threshold of ≥ 0.70.

## Discussion

Through this study, we translated the PCPCM into a Simplified Chinese version specifically tailored for patients receiving family doctor contract services. Necessary adjustments were made, and its psychometric performance was rigorously tested. However, the most substantial challenge in this endeavor was not merely the translation of the PCPCM into Chinese, but achieving a unified understanding of the core theories underlying the functional features of primary care within the mainland Chinese healthcare context. In this setting, primary care gatekeeping is not yet established, and residents lack a consistent habit of seeking care from primary care facilities [25]. Addressing this challenge required identifying the appropriate target population for the translated PCPCM, accurately determining which functional features could realistically be experienced by this population, and clarifying how these choices would impact both the content of the original PCPCM and its application in assessing primary care within the local context.

This challenge led us to thoroughly consider the relationship between the functional features of primary care, service modalities, and the adequate population in mainland China before starting the translation work. Theoretically, the performance and value of primary care are more likely dependent on the overall impact of its functional features and practices rather than the clinical practice of individual diseases [1]. However, the current official metrics for evaluating primary care facilities and family doctor contract services in mainland China often emphasize process and outcome indicators for specific clinical and public health services [26, 27], thereby inadvertently downplaying the crucial role of primary care functions. Recent studies focusing on Chinese residents, especially those with chronic conditions, have revealed significant links between local primary care’s functional features — such as accessibility and continuity — and improved health status [28–30]. These findings provided a crucial foundation for our efforts to translate and validate the PCPCM as an instrument for evaluating the experiences of appropriate “primary care patients” with “primary care services” in mainland China.

In mainland China, the definition of “primary care patients” differs from that in the United States, primarily due to the absence of primary care gatekeeping [31]. Consequently, over 40% of community residents rarely use primary care facilities or engage with genuine primary care services [25, 32]. Furthermore, primary care in China is predominantly provided by government-managed public facilities, such as urban community health service centers/stations and rural township health centers/village health stations [33]. Similarly, the term “clinic patients” in China does not correspond to “primary care patients,” as it often refers to individuals visiting private clinics, which are not the primary providers of primary care services in mainland China [34]. Consequently, retaining terms from the original PCPCM, such as “my clinic” or “my practice,” or targeting all community residents as survey respondents, poses challenges. It can lead to misunderstandings among respondents and limit the instrument’s capacity to accurately capture the functional features of primary care in the local context.

Patients inclined to choose primary care facilities for their health needs represent a broader and more inclusive spectrum of primary care patients. However, it’s crucial to recognize that the distinction in practice between doctors at primary care facilities and general hospitals in mainland China is not as pronounced as the difference between family physicians (general practitioners) and specialists in U.S. and Commonwealth countries [8, 25, 35]. Additionally, the lack of primary care gatekeeping, the focus of medical consultations primarily on treating illnesses rather than providing holistic care, the inability of patients to consistently see the same doctor and establish stable doctor-patient relationships, and the absence of an efficient referral mechanism between primary and secondary care all hinder this population’s ability to effectively and deeply understand the manifestation and value of functional features in these settings [8, 34, 35]. For example, in this study, we had to modify Item 5 of the original PCPCM to better align with local conditions, making it easier for respondents to understand and answer. However, this adjustment may lead to higher scores for Item 5 and the overall average. The revised version asks whether “my family doctor knows about the healthcare services I receive at other institutions,” which sets a lower threshold compared to the original question, “my practice coordinates the care I get from multiple places.”

Ultimately, in developing the PCPCM-SC-FDCS, we specifically targeted patients receiving family doctor contract services at primary care facilities. This selection ensures that the chosen population theoretically has experience with more comprehensive features of primary care—particularly those related to the doctor-patient relationship and community-oriented care, such as continuity and community orientation—allowing them to better understand the content of the PCPCM and provide meaningful responses [13, 17]. However, this choice carries significant implications: it substantially reduces the likelihood of respondents providing negative answers to Items 1 and 2. Within the current structure of the healthcare system in mainland China, residents who lack trust in their family doctor’s ability to effectively treat their illnesses, or who face difficulties accessing their family doctor, are less likely to sign a service contract. Even among those who do sign, they are unlikely to maintain contact or actively seek care, making it challenging for family doctors to engage these individuals or invite them to participate in surveys [9, 36]. Moreover, individuals without actual experience using contracted family doctor services—whether due to a lack of contact or no visits in the past 12 months—are also excluded from the survey population [24]. This exclusion likely results in the PCPCM-SC-FDCS focusing on a narrower subset of the population, one that may report significantly higher levels of functional features compared to primary care patients in countries with established gatekeeping systems. A similar trend was observed in studies using the Traditional Chinese version of the PCPCM in Hong Kong [19].

Psychometric analyses indicate that the PCPCM-SC-FDCS performs well overall. The findings for structural validity, internal consistency, and criterion validity met relevant guideline requirements and were comparable to data from the original and Traditional Chinese versions (Hong Kong) of the PCPCM [13, 19]. The only notable difference was in stability reliability, which was slightly lower than the 0.62 reported for the Traditional Chinese version [19]. Additionally, its excellent feasibility makes it particularly well-suited for busy family doctors, enabling them to quickly and efficiently invite patients to provide immediate assessments of the functional aspects of primary care they have experienced during routine consultations [13]. However, the PCPCM-SC-FDCS has three notable limitations that warrant attention.

First, the cross-sectional design of this study did not allow for the analysis of three critical properties recommended by guidelines [23]: responsiveness, measurement error, and hypothesis testing. Future research should address these gaps through longitudinal cohort studies or randomized controlled trials.

Second, the absence of primary care gatekeeping in China limits the feasibility of evaluating both the accessibility of primary care for the general population and the intensity of functional features experienced by primary care patients within a single survey instrument. When the PCPCM-SC-FDCS focuses exclusively on patients who can effectively and stably access family doctor contracted services, it potentially excludes individuals who voluntarily choose not to engage with primary care. This exclusion may lead to inflated findings that fail to reflect the authentic primary care experiences of the broader population, rendering the results unsuitable for direct comparisons with data from other countries.

Third, the PCPCM’s dimensions, primarily derived from theoretical summaries and reflections of primary care systems and services in the United States [37], may not fully capture the unique functional features of China’s primary care system and family doctor contract services. This limitation is particularly relevant for economically underdeveloped rural populations and urban-rural migrant populations in mainland China, as their economic circumstances, living environments, and healthcare-seeking behaviors often differ significantly from those in the U.S. and Commonwealth countries [8, 38, 39]. These differences suggest the need to refine or develop new instruments that can overcome the inherent limitations of the PCPCM-SC-FDCS, ensuring a more comprehensive and accurate evaluation of primary care in China’s unique healthcare context.

## Conclusion

Our study demonstrates that the PCPCM-SC-FDCS is a reliable and feasible patient-reported experience measure. It has the potential to serve as a valuable tool for assessing primary care functions delivered through family doctor contract services from the perspective of patients in mainland China. However, given the unique context of China’s primary care system, further refinements are necessary. Future efforts should focus on developing more precise measures of primary care accessibility and process quality, identifying and integrating functional features critical to local primary care, and conducting rigorous longitudinal validation studies to strengthen the instrument’s reliability and applicability.

## Electronic supplementary material

Below is the link to the electronic supplementary material.

Supplementary Material 1

## Data Availability

The datasets used and/or analyzed during the current study are available from the corresponding author on reasonable request.
