# Correction: Translation, adaptation, and validation of person-centered primary care measures for patients in family doctor contract services within Mainland China

**DOI:** 10.1186/s12875-025-02857-3

**Published:** 2025-05-10

**Authors:** Yang Wang, Dehua Yu, Hua Jin

**Affiliations:** 1 Department of General Practice, Research Center for General Practice, Yangpu Hospital, School of Medicine, Tongji University, Shanghai, China; 2 Shanghai General Practice and Community Health Development Research Center, Shanghai, China

**Correction: BMC Prim. Care 26**,** 91 (2025)**

10.1186/s12875-025-02796-z

Following publication of the original article [1], the author reported that the affiliation 1 was incorrectly presented. Below is the correct affiliation presentation.

Incorrect:

^1^Department of General Practice, Research Center for General Practice, School of Medicine, Yangpu Hospital, Tongji University, Shanghai, China

Correct:

^1^Department of General Practice, Research Center for General Practice, Yangpu Hospital, School of Medicine, Tongji University, Shanghai, China

The original article [1] has been corrected.
