# Supplementary material for: Translation, adaptation, and validation of Person-Centered Primary Care Measures for patients in family doctor contract services within mainland China
Source: BMC Prim Care. 2025 Mar 31;26:91. doi: 10.1186/s12875-025-02796-z (PMC11956412; doi:10.1186/s12875-025-02796-z)
Supplement: Supplementary file 1 — Supplementary Material 1 [file 12875_2025_2796_MOESM1_ESM.docx]

**Table S1 Preliminary Simplified Chinese versions of the PCPCM**

| Original Version | Pre-final versions of PCPCM-SC-FDCP | Back-Translation Version |
| --- | --- | --- |
| How would you assess your primary care experience? | 你如何评价你的家庭医生签约服务体验？ | How would you assess your experience with the family doctor contract service? |
| My practice makes it easy for me to get care. | 我签约的团队能让我方便地得到医疗服务 | The team I signed up with makes it easy for me to get medical care. |
| My practice is able to provide most of my care. | 我签约的团队能提供我所需的大部分医疗服务 | The family doctor team I signed up with is able to provide most of medical care I need. |
| In caring for me, my doctor considers all factors that affect my health. | 我的家庭医生在治疗我时，会考虑所有可能影响我健康的因素 | In caring for me, my family doctor considers all factors that affect my health. |
| My practice coordinates the care i get from multiple places. | 我签约的团队能帮我综合管理我在不同地方接受的医疗服务 | The family doctor team I signed up with integrate and manage the medical care I get from multiple places. |
| My doctor or practice knows me as a person. | 我的家庭医生或团队很了解我这个人 | My family doctor or the team knows me as a person. |
| My doctor and i have been through a lot together. | 我和我的家庭医生一起经历过很多事情 | I have been through a lot with my family doctor. |
| My doctor or practice stands up for me. | 我的家庭医生或团队会维护我的权益 | My family doctor stands up for my benefits. |
| The care i get takes into account knowledge of my family. | 我接受过的家庭医生的医疗服务考虑到了我家庭的情况 | The care I receive from my family doctor takes into account the situation of my family. |
| The care i get in this practice is informed by knowledge of my community. | 我接受过的家庭医生的医疗服务考虑到了我社区的情况 | The care I receive from my family doctor takes into consideration the information of my community. |
| Over time, my practice helps me to stay healthy. | 随着时间的推移，我签约的团队帮助我保持健康 | Over time, the team I've contracted with helps me stay healthy. |
| Over time, my practice helps me to meet my goals. | 随着时间的推移，我签约的团队帮助我达到我的健康目标 | Over time, the team I've contracted with helps me reach my health goals. |
| Definitely | 确实是这样 | Definitely |
| Mostly | 多数时候是这样 | Most of the time |
| Somewhat | 有时候是这样 | Sometimes |
| Not at all | 完全不是这样 | Not at all. |

**Table S2 Results of Pilot Testing of PCPCM-SC-FDCP**

| Original version | Pre-final versions | Patients' Clarity Ratings for Pre-Final Versions | Experts' Clarity Ratings for Pre-Final Versions | Revised Version for Testing | Clarity Ratings for Revised Version (Patients/Experts) | S-CVI/Ave |
| --- | --- | --- | --- | --- | --- | --- |
| **How would you assess your primary care experience?** | 你如何评价你的家庭医生签约体验？ | 100% | 100% | No revised | - |  |
| **My practice makes it easy for me to get care.** | 我签约的团队能让我方便地得到医疗服务 | 95% | 70% | 我签约的家医团队能让我方便地得到医疗保健服务 | 100%/100% | 1 |
| **My practice is able to provide most of my care.** | 我签约的团队能提供我所需的大部分医疗服务 | 100% | 70% | 我签约的家医团队能为我提供我所需的大部分医疗保健服务 | 100%/100% | 0.9 |
| In caring for me, my doctor considers all factors that affect my health. | 我的家庭医生在治疗我时，会考虑所有可能影响我健康的因素 | 90% | 70% | 我的家庭医生在治疗我时，考虑到了所有可能影响我健康的因素(如心理压力、饮食健康、家庭关系等) | 100%/100% | 0.9 |
| My practice coordinates the care I get from multiple places. | 我签约的团队能帮我综合管理我在不同地方接受的医疗服务 | 50% | 30% | 我的家庭医生知道我在其它医疗机构接受的医疗保健服务 | 100%/100% | 0.9 |
| My doctor or practice knows me as a person. | 我的家庭医生或团队很了解我这个人 | 80% | 80% | 我的家庭医生或团队很了解我这个人的情况 | 100%/100% | 0.8 |
| 0.9My doctor and I have been through a lot together. | 我和我的家庭医生一起经历过很多事情 | 90% | 90% | No revised | - | 0.9 |
| My doctor or practice stands up for me. | 我的家庭医生或团队会维护我的权益 | 80% | 80% | 我的家庭医生或团队会维护我的利益 | 90%/90% | 1 |
| The care I get takes into account knowledge of my family. | 我接受过的家庭医生的医疗服务考虑到了我家庭的情况 | 100% | 100% | No revised | - | 0.9 |
| The care I get in this practice is informed by knowledge of my community. | 我接受过的家庭医生的医疗服务考虑到了我社区的情况 | 100% | 100% | No revised | - | 0.8 |
| Over time, my practice helps me to stay healthy. | 随着时间的推移，我签约的团队帮助我保持健康 | 85% | 80% | 在过去一段时间里，我签约的家医团队帮助我维持良好的健康状态 | 90%/90% | 0.9 |
| Over time, my practice helps me to meet my goals. | 随着时间的推移，我签约的团队帮助我达到我的健康目标 | 85% | 80% | 在过去一段时间里，我签约的家医团队帮助我实现我的健康目标 | 90%/90% | 0.9 |
| Definitely | 确实是这样 | 100% | 80% | 确实这样 | 100%/100% |  |
| Mostly | 多数时候是这样 | 100% | 80% | 基本这样 | 100%/100% |  |
| Somewhat | 有时候是这样 | 100% | 80% | 或许这样 | 100%/100% |  |
| Not at all | 完全不是这样 | 100% | 80% | 不是这样 | 100%/100% |  |

**Note:**

**S-CVI/Ave:** Scale-Content Validity Index/Average
